# Development of a Nomogram for Predicting Refractory *Mycoplasma pneumoniae* Pneumonia in Children

**DOI:** 10.3389/fped.2022.813614

**Published:** 2022-02-25

**Authors:** Fangfang Shen, Chunjuan Dong, Tongqiang Zhang, Changjiang Yu, Kun Jiang, Yongsheng Xu, Jing Ning

**Affiliations:** Department of Pediatric Respiratory Medicine, Tianjin Children's Hospital, Tianjin University Children's Hospital, Tianjin, China

**Keywords:** *Mycoplasma pneumoniae* pneumonia, refractory, nomogram, CRP, LDH, D-dimer

## Abstract

**Background:**

In children, refractory *Mycoplasma pneumoniae* pneumonia (RMPP) may result in severe complications and high medical costs. There is research on a simple and easy-to-use nomogram for early prediction and timely treatment of RMPP.

**Methods:**

From December 2018 to June 2021, we retrospectively reviewed medical records of 299 children with *Mycoplasma pneumoniae* pneumonia (MPP) hospitalized in Tianjin Children's Hospital. According to their clinical manifestations, patients were divided into the RMPP group and the general *Mycoplasma pneumoniae* pneumonia (GMPP) group. The clinical manifestations, laboratory indicators, and radiological data of the two groups were obtained. Stepwise regression was employed for variable selection of RMPP. The predictive factors selected were used to construct a prediction model which presented with a nomogram. The performance of the prediction model was evaluated by C statistics, calibration curve, and receiver operating characteristic (ROC) curve.

**Results:**

The RMPP group significantly showed a higher proportion of females, longer fever duration, and longer hospital stay than the GMPP group (*P* < 0.05). Additionally, the RMPP group revealed severe clinical characteristics, including higher incidences of extrapulmonary complications, decreased breath sounds, unilateral pulmonary consolidation >2/3, and plastic bronchitis than the GMPP group (*P* < 0.05). The RMPP group had higher neutrophil ratio (N%), C-reactive protein (CRP), interleukin-6 (IL-6), lactic dehydrogenase (LDH), and D-dimer than the GMPP group (*P* < 0.05). Stepwise regression demonstrated that CRP [OR = 1.075 (95% CI: 1.020–1.133), *P* < 0.001], LDH [OR = 1.015 (95% CI: 1.010–1.020), *P* < 0.001], and D-dimer [OR = 70.94 (95% CI: 23.861–210.904), *P* < 0.001] were predictive factors for RMPP, and developed a prediction model of RMPP, which can be visualized and accurately quantified using a nomogram. The nomogram showed good discrimination and calibration. The area under the ROC curve of the nomogram was 0.881, 95% CI (0.843, 0.918) in training cohorts and 0.777, 95% CI (0.661, 0.893) in validation cohorts, respectively.

**Conclusion:**

C-reactive protein, LDH, and D-dimer were predictive factors for RMPP. The simple and easy-to-use nomogram assisted us in quantifying the risk for predicting RMPP, and more accurately and conveniently guiding clinicians to recognize RMPP, and contribute to a rational therapeutic choice.

## Introduction

In recent years, *Mycoplasma pneumoniae* (MP)-related respiratory tract infections in children have increased significantly worldwide, and 10–40% of these infections will develop into *Mycoplasma pneumoniae* pneumonia (MPP) (1–3), of which pleural effusion, plastic bronchitis, and atelectasis are all possible complications during the acute stage, as are necrotizing pneumonia, pulmonary thromboembolism, lung abscess, and other severe complications (4, 5). In recent years, the number of cases of MPP complicated with thromboembolism has increased (6). Some children had poor responses to conventional MPP treatment, presenting as refractory *Mycoplasma pneumoniae* pneumonia (RMPP). Children with RMPP, which may be life-threatening, with multiple systems and organ involvement such as nervous, digestive, blood, and mucocutaneous systems, may have sequelae (7, 8). Chronic pneumonia, recurrent respiratory tract infections, and asthma with RMPP have caused great pain to patients, increased medical burden, and posed great challenges for clinicians (9). This hot spot, difficult issue is more prominent in Asia, particularly in China. Therefore, clinicians should pay more attention to clinical characteristics and relevant laboratory indicators, allowing for early RMPP prediction and intervention.

Refractory *Mycoplasma pneumoniae* pneumonia pathogenesis remains unclear. Multiple factors contribute to the difficulty of treating RMPP. Numerous studies indicate that prolonged fever lasting more than 10 days, C-reactive protein (CRP), lactic dehydrogenase (LDH), neutrophil ratio (N%), ferritin (FER), interleukin-10 (IL-10), IL-18, tumor necrosis factor (TNF-α), interferon-gamma (INF-γ), CARDS toxin, and macrolide-resistant are risk factors for RMPP (10–14). The statistical method for single-factor analysis of RMPP has certain limitations, and comprehensive analysis should be conducted combining clinical manifestations, laboratory indicators, and radiological data. In addition, clinicians who have difficulty in detecting cytokines and other indicators, urgently need a simple and easy method to predict RMPP using clinically accessible data. Due to their good predictive ability and simplicity, nomograms are widely employed in diagnosing diseases. However, there is currently little research on nomograms for RMPP prediction.

In this study, a case-control study was conducted to comprehensively analyze clinical manifestations, laboratory indicators, and radiological data of 299 children with MPP to identify significant predictors for RMPP. Using readily available clinical data, we established a nomogram to predict RMPP. The nomogram visualizes and quantifies the prediction indicators of RMPP. It is straightforward and easy to use and can contribute to early detection and timely treatment of RMPP, reducing the risk of serious complications and medical burdens.

## Methods

### Study Population and Data Collection

This retrospective study was a single-center clinical data analysis. This study was exempted from ethical approval procedures because no clinical trials or randomized controlled trials were conducted, and individual consent was waived.

From December 2018 to June 2021, we retrospectively collected and analyzed data of children with pneumonia hospitalized in Tianjin Children's Hospital. Inclusion criteria were: (1) hospitalized patients under 18 years old; (2) symptoms and signs indicative of community-acquired pneumonia (CAP), including fever, cough, abnormal lung auscultation (lung rale and decreased breath sounds), and new infiltration on chest radiograph; (3) had positive result for MP. The diagnosis of MP infection was based on both of two positive laboratory results, including MP-immunoglobulin M (IgM) titer ≥1:160 or four-fold rising titer in acute and convalescent serum specimens (interval of 2 weeks), and positive results of MP polymerase chain reaction (PCR) in nasopharyngeal secretions (15). Refractory *Mycoplasma pneumoniae* pneumonia diagnosis was based on diagnosing MPP in patients treated with macrolide antibiotics for 7 days or more, with prolonged fever (>38.5°C), deterioration of clinical symptoms (violent coughing, wheezing, chest pains, and trouble breathing), and progressive pulmonary radiological examination exacerbation (the scope of lung lesion expands, density increases, pleural effusion, even necrotizing pneumonia, and lung abscess pneumonia) (16). The MPP patients were divided into two groups: general *Mycoplasma pneumoniae* pneumonia (GMPP) group and RMPP group. Exclusion criteria included: (1) patients with immunodeficiency disease, chronic pulmonary disease, cardiac disease or chronic glomerulonephritis, rheumatic disease, malnutrition, diabetes, and other genetic metabolic diseases; (2) patients co-infected with other pathogens and tuberculosis; (3) patients in their recoveries from MPP, and (4) patients who had incomplete clinical records.

Demographic, clinical, laboratory, and radiological characteristics were collected from medical histories. Demographic data included age and sex. Clinical characteristics included the duration of fever (time from the onset of fever to the date of hospitalization), length of stay in the hospital, cough, wheezing, extrapulmonary complications (hepatic injury, rash, myocarditis, encephalitis or encephalopathy, albuminuria, hematuria, and hemolytic anemia), and pulmonary signs (lung rale and decreased breath sounds). At admission, laboratory indicators of the two groups included white blood cell (WBC), N%, platelet counts (PLT), CRP, LDH, IL-6, FER, erythrocyte sedimentation rate (ESR), D-dimer, prealbumin (Pa), and procalcitonin (PCT). All patients underwent chest radiography during their illness. When severe intrapulmonary complications occurred, the radiologist assessed for atelectasis, unilateral pulmonary, consolidation >2/3, pulmonary necrosis, pleural effusion, and pulmonary embolism by chest CT scans. The radiological data (atelectasis, unilateral pulmonary consolidation >2/3, pulmonary necrosis, pleural effusion, and pulmonary embolism) and fiber optic bronchoscopy data of the two groups were collected. Indication of fiber optic bronchoscopy according to Chinese guidelines and standards: restoring airway patency in cases of lobar consolidation or atelectasis persisted on chest X-ray film after proper therapy for 1 week (15, 17–19). Bronchial mucus plugs were discovered in the lungs of children who required fiber optic bronchoscopy and bronchoalveolar lavage. The bronchial mucus plugs reported to be composed of mucin and/or fibrin, were placed under physiological saline and presented a bronchial tree tube, pathologic diagnosis as plastic bronchitis.

### Statistical Analysis

Excel software was used to input the above-mentioned data, a double data entry was adopted. As for the quantitative data, mean ± standard deviation ($\bar{x}\pm s$) or median and quartile [M (P25, p75)] was used in descriptive statistics and a *T*-test or Mann-Whitney rank-sum test was performed in the comparisons between groups as appropriate. The counting data was expressed as a percentage (%), and Chi-square test was used to compare discrepancy between groups. Stepwise regression was employed for variable selection. The predictive factors selected were used to construct a prediction model which presented with a nomogram. We used bootstrapping validation (1,000 bootstrap resamples) for internal validation. The accuracy of the nomogram was assessed using the discrimination ability (C statistics) and the calibration curve. C statistic is equivalent to the area under the receiver operating characteristic (ROC). Calibration curve was evaluated with unreliability U test in both training cohorts and validation cohorts. Statistical significance was defined as *P* < 0.05. All statistical analyses were performed using SPSS version 22.0. Model building and diagram drawing were performed using R software (version 3.4.3; http://www.r-project.org).

## Results

### Clinical Characteristics

A total of 334 patients were screened. We excluded 35 patients, including nine patients with diseases of exclusion criteria (1), 22 patients co-infected with other pathogens, three patients in their recoveries from MPP, and one patient who had incomplete clinical records. There were 299 patients with MPP enrolled, including 156 children in the RMPP group, and 143 children in the GMPP group. A total of 299 children were selected as the training cohort. In Table 1, it is found that in terms of demographic and clinical characteristics of children with MPP, there are statistically significant differences between RMPP and GMPP groups in sex, fever duration, length of stay in hospital, extrapulmonary complications, and decreased breath sounds in pulmonary signs. The RMPP group had a significantly higher proportion of females, a longer fever duration, and longer hospital stay than the GMPP group (*P* < 0.05). Fever duration, length of stay in hospital, decreased breath sounds, and extrapulmonary complications in the RMPP group are significantly higher than those in the GMPP group (*P* < 0.05). In Table 2, compared with the GMPP group, the RMPP group had higher levels of N% (73.1 vs. 56.6%), CRP (22.48 vs. 18.55 mg/L), IL-6 (44.8 vs. 25.2 pg/mL), LDH (439.69 vs. 392.33 U/L), and D-dimer (3.75 vs. 3.21 mg/L) (*P* < 0.05). A total of 33 cases in the RMPP group and 28 cases in the GMPP group conformed to the criteria of fiber optic bronchoscopy and alveolar lavage. In Table 3, the RMPP group also revealed severe radiological data and fiber optic bronchoscopy data, as well as higher incidences of unilateral pulmonary consolidation >2/3 and plastic bronchitis than the GMPP group (*P* < 0.01).

**Table 1:** Clinical characteristics of GMPP and RMPP patients.

**Clinical characteristic**	**RMPP**	**GMPP**	***P***
Sex (male) [*n* (%)]	55 (35.2%)	70 (49.0%)	0.023
Age (years)	6.80 ± 2.30	7.20 ± 2.20	0.126
Duration of fever (days)	12.37 ± 3.42	8.16 ± 2.34	<0.001
Length of stay in hospital (days)	11.24 ± 2.34	7.62 ± 1.98	<0.001
Clinical symptoms			
Cough [*n* (%)]	156 (100%)	143 (100%)	1.000
High fever [*n* (%)]	156 (100%)	114 (79.7%)	1.000
Wheezing [*n* (%)]	10 (0.06%)	12 (0.08%)	0.342
Extrapulmonary complications [*n* (%)]	62 (39.7%)	20 (13.99%)	<0.001
Pulmonary signs			
Lung rale [*n* (%)]	93 (59.6%)	100 (69.9%)	0.081
Decreased breath sound [*n* (%)]	54 (34.6%)	26 (17.44)	<0.001

**Table 2:** Laboratory data of GMPP and RMPP.

	**RMPP**	**GMPP**	***p***
*n*	156	143	
WBC (×10^9^/L)	6.57 [5.03, 8.23]	6.89 [6.08, 9.12]	0.632
N%	73.10 [68.97, 76.35]	56.60 [52.70, 59.25]	<0.001
PLT (×10^9^/L)	529.30 [509.95, 547.03]	533.80 [518.50, 547.25]	0.274
CRP (mg/L)	22.48 [17.41, 28.59]	18.55 [16.20, 25.36]	<0.001
LDH (U/L)	439.69 [391.72, 497.88]	392.33 [329.01, 446.97]	<0.001
IL-6 (pg/ml)	44.8[27.1–78.2]	25.2[14.1–38.4]	<0.001
FER (ng/ml)	233.10 [216.05, 252.47]	232.60 [210.10, 243.25]	0.072
ESR (mm/h)	13.90 [12.15, 16.50]	14.50 [12.70, 16.75]	0.388
D-dimer (mg/L)	3.75 [3.48, 3.95]	3.21 [3.00, 3.62]	<0.001
Pa (g/L)	0.15 [0.14, 0.16]	0.16 [0.14, 0.17]	0.063
PCT (ng/ml)	0.15 [0.13, 0.17]	0.15 [0.12, 0.18]	0.874

*WBC, White blood cell; N%, neutrophil ratio; PLT, platelet counts; CRP, C-reactive protein; LDH, lactic dehydrogenase; IL-6, interleukin-6; FER, ferritin; ESR, erythrocyte sedimentation rate; D-dimer; Pa, prealbumin; PCT, procalcitonin*.

**Table 3:** Radiological data and fiberoptic bronchoscopy data of GMPP and RMPP patients.

	**GMPP**	**RMPP**	***P***
Atelectasis	54	74	0.091
Unilateral pulmonary consolidation >2/3	90	124	0.002
Pulmonary necrosis	6	9	0.509
Pleural effusion	38	46	0.571
Plastic bronchitis	12	30	0.008
Pulmonary embolism	0	1	0.965

### Selecting Predictive Factors by Stepwise Regression

The clinical variables associated with RMPP were assessed based on clinical importance and predictors identified in previously published articles, and the indexes with statistically significant differences were selected from Tables 1–3 as the included variables as well. Stepwise regression (forward, backward, back-and-forth selection) was used for variable selection. The variables corresponding to the smallest AIC value were selected. Finally, CRP [OR = 1.075 (95% CI: 1.020–1.133), *P* < 0.001], LDH [OR = 1.015 (95% CI: 1.010–1.020), *P* < 0.001], and D-dimer [OR = 70.94 (95% CI: 23.861–210.904), *P* < 0.001] were selected as three predictive factors for RMPP (Table 4).

**Table 4:** Predictive factors for RMPP by stepwise regression.

**Intercept and variable**	**β Coefficient**	**OR (95%CI)**	***P*** **-value**
D-dimer	4.262	70.940 (23.861–210.9044)	*P* <0.001
CRP	0.073	1.075 (1.020–1.133)	*P* <0.001
LDH	0.015	1.015 (1.010–1.020)	*P* <0.001
Constant	−22.876	NA	NA

*CI, confidence interval; NA, not applicable; OR, odds ratio*.

### Development and Validation of Nomogram

**Figure 1:**
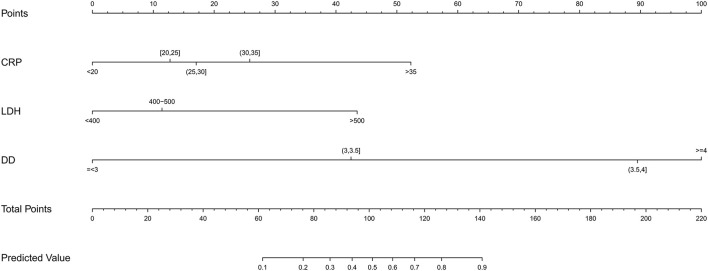
Construct a nomogram of risk for RMPP children. A nomogram predicting the risk of RMPP was established based on the three independent risk factors (CRP level, D-dimer level, and LDH level). You can specify points for each variable by drawing a line up from the corresponding variable to the point line. The sum of the points plotted on the Total Points line and the risk value corresponding to the RMPP risk score represented the specific risk at which the RMPP occurred.

According to the selected variables, the nomogram of prediction model with RMPP was constructed (Figure 1), which can be visualized and accurately quantified. The sum of corresponding scores of CRP, LDH, and D-dimer in this nomogram was associated with an increased risk of RMPP. Taking the laboratory indicators of a child infected with MP as an example, CRP, LDH, and D-dimer were 26 (mg/L), 388 (U/L), and 3.3 (mg/L), respectively. The child's nomogram scores are 12, 0, and 42, for a sum of 54. According to the probability of RMPP corresponding to the total score at the chart's bottom, the probability of RMPP was <10%. The nomogram had good accuracy and discrimination ability for prediction because of its objectivity and impartiality.

#### Validation

**Figure 2:**
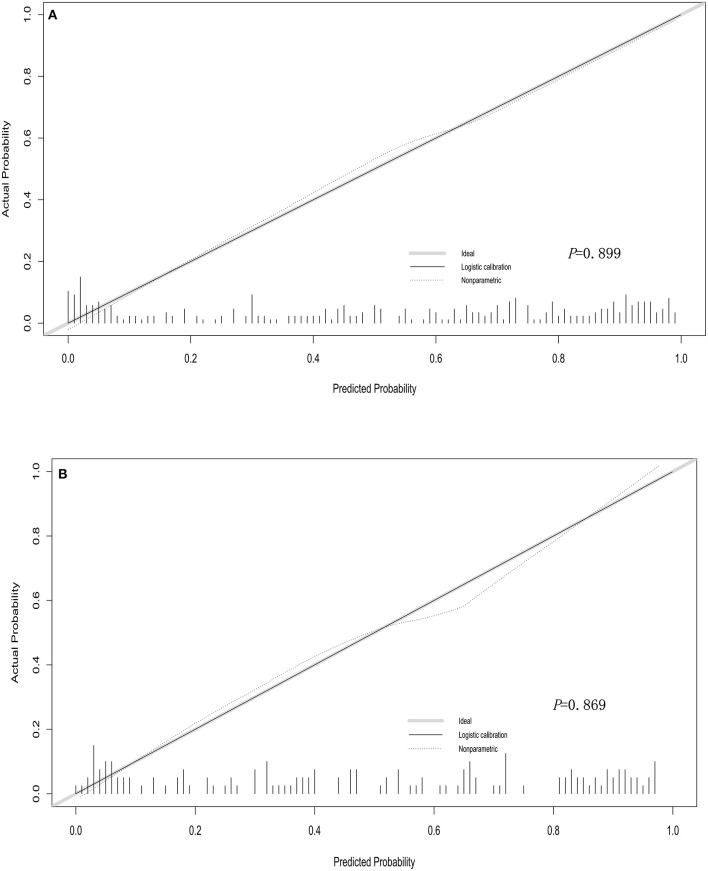
Calibration curves of the nomogram in training cohorts **(A)** and validation cohorts **(B)**. The calibration curve predicts the risk of RMPP in the queue. The x-axis represented the projected risk of RMPP. The y-axis represented the RMPP of the actual diagnosis. The diagonal dotted line represented the perfect prediction of the ideal model. The solid line represented the performance of the line chart, which is closer to the diagonal dashed line to indicate a better prediction.

**Figure 3:**
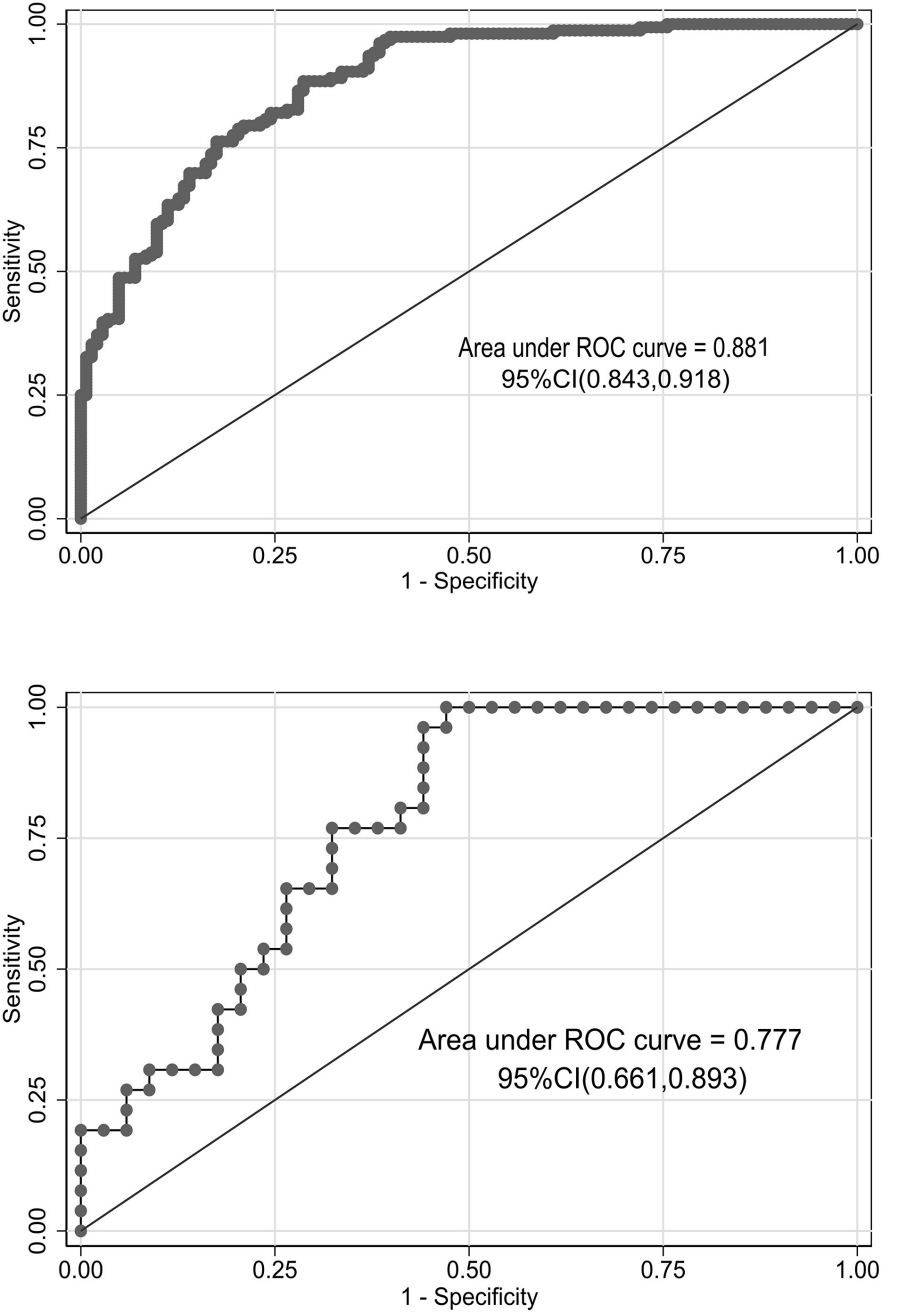
Receiver operating characteristic (ROC) curves of the nomograms in training and validation cohorts. The area under the ROC curve in the training cohorts and the validation cohorts was 0.881 (95% CI: 0.843–0.918) and 0.777 (95% CI: 0.661–0.893), respectively.

We used bootstrapping validation (1,000 bootstrap resamples) for internal validation. C statistics in the training and validation cohorts were 0.878, 95% CI (0.841, 0.915) and 0.787, 95% CI (0.750, 0.824), respectively. The calibration curves of the nomogram showed good probability consistencies between prediction and observation in the training cohorts (*P* = 0.899) (Figure 2A) and validation cohorts (*P* = 0.869) (Figure 2B). The areas under the ROC curve of the probability of RMPP were 0.881 (95% CI: 0.843–0.918) in the training cohorts and 0.777 (95% CI: 0.661–0.893) in the validation cohorts (Figure 3).

## Discussion

While most children with MPP have a good prognosis after macrolides treatment, those with RMPP and severe MPP have poor outcome on macrolides treatment and prolonged course of the disease. Not only are numerous complications present during the acute stage, but lung structure or function may be compromised, resulting in long-term complications, such as post-infectious bronchiolitis obliterans, bronchial asthma, unilateral transparent lung, bronchiectasis, etc. (20, 21). The main objective of this study was to analyze predictive factors for RMPP and establish an easy-to-use nomogram for RMPP prediction for early detection and timely treatment to avoid complications and sequelae.

The complex pathogenesis of RMPP in children remains unknown. It is usually a comprehensive effect of multiple factors, which are intimately related to the direct pathogenesis of mycoplasma and disorder of the body's immune response. Current studies suggest that MP infects the body and adheres to respiratory epithelial cells, induces respiratory epithelial adhesion protein expression, and significantly increases airway mucus secretion and formation of bronchial mucus plugs. The plastic bronchitis results in the appearance of pulmonary signs such as deceased breath sound and radiological evidence of atelectasis or segmental pulmonary consolidation (22). Simultaneously, following MP infection, the immune function of several links between innate and adaptive immunity was disordered, leading to excessive inflammation in the lungs and the whole body (23). Cytokines and chemokines released during these hyperinflammatory responses produce inflammatory cascade amplifications (24). Numerous studies have revealed an increase in Il-18, Il-6, TNF-α, and Il-10 levels in children with RMPP (12, 25). The cytokine storm promotes neutrophil infiltration (26). Further aggravation of lung lesions, necrotizing pneumonia, pleural effusion, and extrapulmonary complications resulted in prolonged fever and hospitalization in the RMPP group (14, 27). Our analysis revealed that the RMPP group had decreased breath sounds, unilateral pulmonary consolidation >2/3 radiologically, and higher levels of N% and IL-6 than the GMPP group, the incidence of plastic bronchitis, extrapulmonary complications, and length of stay in the hospital were significantly higher than the GMPP group, with statistically significant differences. Furthermore, stepwise regression revealed that CRP, LDH, and D-dimer were predictive factors for RMPP and were included in the nomogram.

C-reactive protein is the most frequently used marker of acute phase non-specific inflammation. C-reactive protein eliminates pathogenic microorganisms and damaged, necrotic, and apoptotic tissue cells by activating complement and strengthening the phagocytosis of phagocytes. C-reactive protein begins to rise 2–8 h after infection-induced inflammation or tissue damage, peaks at 24–48 h, and rapidly returns to normal levels as lesions subside and tissue structure and function recover. A study found that dynamic monitoring of CRP might be more effective at capturing progression and evaluating CAP prognosis (28). Our study found that CRP levels were statistically significantly higher in the RMPP group than in the GMPP group, CRP was a predictive factor for RMPP, and monitoring changes in CRP elevation can assist in early detection of RMPP. Similar to our study, CRP > 40 mg/L is a risk factor for early evaluation and identification of RMPP: the higher the detected value, the more risk of having RMPP (14, 29).

Lactic dehydrogenase is a readily available non-specific inflammatory biomarker of tissue damage and is present in the cytoplasm of all tissues. It is released into the serum when cells dissolve or cell membranes are damaged and serves as an important indicator to monitor infection severity and inflammatory disease. In this study, stepwise regression revealed that LDH was a predictive factor for RMPP. Excessive inflammatory responses in the lung, both local and systemic, in children with RMPP led to increased cell membrane permeability, resulting in increased LDH. Research has indicated that LDH isoenzymes 4 and 5 in LDH are indicators for predicting RMPP severity in children (30). However, there is currently no consensus on the critical value of LDH for RMPP warning (22, 31). However, numerous studies in recent years indicate that LDH levels are significantly elevated in children with RMPP, which often indicates the need for early glucocorticoid therapy. Lactic dehydrogenase can be used as a marker of glucocorticoid therapy for RMPP (12). Additionally, a recent study found that LDH levels at admission were associated with slow response to MPP treatment and no response or progression of MPP (32).

D-dimer is a terminal product formed when a fibrinolytic enzyme acts on crosslinked fibrin. It is an independent predictor of risk of thrombotic events. D-dimer is a molecular marker of hypercoagulable state and secondary hyperfibrinolysis of the body, which has been widely applied to exclude thromboembolism, particularly acute pulmonary embolism diagnosis and thrombolysis efficacy evaluation (33). D-dimer has recently been used to assess MPP severity. D-dimer levels are elevated in MPP patients and are linked to MPP severity (34). Elevated D-dimer levels can be utilized as an early predictor of RMPP and complications such as liver injury and pleural effusion (29). Additionally, our study demonstrated that D-dimer is a predictive factor for predicting RMPP, and increased D-dimer levels may be associated with a stronger inflammatory response. The direct infection of MPP and abnormal body immune response lead to systemic inflammation, followed by vascular endothelial injury, subcutaneous collagen exposure, and vasoconstriction. Disrupting the balance of blood coagulation and anticoagulation system causes a hypercoagulable state and higher D-dimer levels. Promoted vasculitis or micro thrombosis contributes to necrosis, pulmonary embolism, and even brain embolism, spleen, and peripheral arteries, resulting in multiple organ failure and high mortality rates. Therefore, elevated D-dimer levels in children with RMPP indicate hypercoagulability and vascular endothelial dysfunction, which correlates with disease severity (35). Elevated D-dimer levels, particularly those >11.1 mg/L, also contributed to the early diagnosis of RMPP with thrombosis (36). A prospective study with a 5-year follow-up discovered that MP infection was independently associated with the risk of developing subsequent ischemic stroke in over 1,000 patients with MP infection (37). In our RMPP group, one child with severe RMPP was monitored on the eighth day of disease onset with D-dimer levels increased to 13 mg/L, and color Doppler echocardiography revealed multiple masses in the right ventricle. Spiral CT pulmonary angiography showed a filling defect in the left lower pulmonary artery and a nodular filling defect in the right ventricle. Right ventricle thrombosis and left lower pulmonary thromboembolism were considered. This child has abnormal hemodynamics, indicating high-risk pulmonary thromboembolism. D-dimer levels returned to normal 4 weeks after onset when glucocorticoid anti-inflammatory therapy was combined with active intravenous thrombolysis and anticoagulant therapy. Three months later, the pulmonary artery and right ventricle thrombosis resolved. After timely administration of anticoagulant therapy, long-term prognosis of thrombosis in children with RMPP was favorable. It is suggested that increased D-dimer levels are related to disease severity and inflammation degree, and D-dimer levels can be reduced to normal levels once inflammation is controlled.

Refractory *Mycoplasma pneumoniae* pneumonia treatment is challenging; the higher the above three indicators, which stimulate the immune response and inflammatory factors stimulation, the more serious the clinical manifestations and tissue pathological changes. The importance of initiating glucocorticoid therapy as soon as possible, rather than waiting for the effect of antibiotics, can reduce immune-mediated injury in MP, improve treatment efficacy, and prevent complications. The differences between GMPP and RMPP in clinical manifestations and laboratory indicators are close and difficult to distinguish, which can easily induce clinical medical workers to either ignore disease severity or administer excessive treatment. Therefore, developing a nomogram that can accurately predict RMPP based on risk factors is critical for clinical diagnosis and treatment. However, the current clinical prediction method is, in essence, a complicated formula calculation in which individual predictors are given varying weights to calculate the probability of disease, and thus is extremely unsuitable for clinical application. However, our study for RMPP prediction presents a simple, intuitive, and scientifically accurate nomogram, in which the three predictive factors of RMPP, CRP, LDH, and D-dimer demonstrate significant predictive value. It can assist clinicians, particularly those working in primary medical institutions, in rapidly calculating the incidence probability and risk of RMPP in children using simple and easy-to-obtain laboratory indicators commonly used in clinics to carry out individualized treatment of RMPP as soon as possible. The nomogram is easy to apply clinically, with good discriminative ability and accuracy and benefit for children. The advantages mentioned above are currently irreplaceable by other clinical tools.

Additionally, our research has some limitations. First, this retrospective study was based on patient data from a 1,700-bed teaching hospital. In the future, it is necessary to increase the number of study populations and centers and incorporate more indicators of possible clinical significance into the nomogram to provide more meaningful assistance with early clinical detection of RMPP. In addition, although we have validated the nomogram internally within the same medical center, more prospective multicenter studies are required for external validation.

In conclusion, CRP, LDH, and D-dimer were independent indicators for RMPP. The simple and easy-to-use nomogram assisted us in quantifying the risk for predicting RMPP, and more accurately and conveniently guiding clinicians to recognize RMPP and contribute to a rational therapeutic choice.

## Data Availability Statement

The original contributions presented in the study are included in the article/supplementary material, further inquiries can be directed to the corresponding author/s.

## Ethics Statement

Written informed consent was not obtained from the minor(s)' legal guardian/next of kin for the publication of any potentially identifiable images or data included in this article.

## Author Contributions

FS, CD, TZ, and CY wrote the main manuscript text and KJ prepared all figures. All authors reviewed the manuscript.

## Conflict of Interest

The authors declare that the research was conducted in the absence of any commercial or financial relationships that could be construed as a potential conflict of interest.

## Publisher's Note

All claims expressed in this article are solely those of the authors and do not necessarily represent those of their affiliated organizations, or those of the publisher, the editors and the reviewers. Any product that may be evaluated in this article, or claim that may be made by its manufacturer, is not guaranteed or endorsed by the publisher.
